# Association of Continuation of Statin Therapy Initiated Before Transition to Chronic Dialysis Therapy With Mortality After Dialysis Initiation

**DOI:** 10.1001/jamanetworkopen.2018.2311

**Published:** 2018-10-05

**Authors:** Elani Streja, Elvira O. Gosmanova, Miklos Z. Molnar, Melissa Soohoo, Hamid Moradi, Praveen K. Potukuchi, Kamyar Kalantar-Zadeh, Csaba P. Kovesdy

**Affiliations:** 1 Harold Simmons Center for Kidney Disease Research and Epidemiology, Division of Nephrology and Hypertension, Department of Medicine, University of California Irvine Medical Center, Orange; 2 Nephrology Section, Tibor Rubin Veterans Affairs Medical Center, Long Beach, California; 3 Nephrology Section, Stratton Veterans Affairs Medical Center, Albany, New York; 4 Division of Nephrology, Department of Medicine, Albany Medical College, Albany, New York; 5 Division of Transplant Surgery, Methodist University Hospital Transplant Institute, Memphis, Tennessee; 6 Department of Surgery, University of Tennessee Health Science Center, Memphis; 7 Department of Transplantation and Surgery, Semmelweis University, Budapest, Hungary; 8 Division of Nephrology, Department of Medicine, University of Tennessee Health Science Center, Memphis; 9 Nephrology Section, Memphis Veterans Affairs Medical Center, Memphis, Tennessee

## Abstract

**Importance:**

De novo statin therapy in patients receiving chronic dialysis has failed to demonstrate cardiovascular (CV) protection in randomized clinical trials and thus is not recommended by current guidelines. However, current guidelines recommend the continuation of statin therapy if initiated before transition to dialysis.

**Objective:**

To investigate whether the continuation of statins from advanced chronic kidney disease into the dialysis therapy period is associated with improved survival.

**Design, Setting, and Participants:**

Retrospective cohort study of US veterans transitioning to dialysis between October 1, 2007, and March 30, 2014. Participants were 14 298 US veterans who were receiving statins during the 12-month period before transition to dialysis and survived the first year of dialysis. Data analysis was conducted between August 2, 2017, and June 28, 2018.

**Exposures:**

Patients were characterized as statin continuers (n = 11 936) if statin therapy was continued for at least 6 months during the first year after dialysis initiation and as statin discontinuers (n = 2362) if therapy with statins was stopped or no statin therapy was received in the year posttransition.

**Main Outcomes and Measures:**

Associations of statin continuation with 12-month all-cause mortality and CV mortality after 1 year of dialysis initiation were examined using Cox proportional hazards regression models adjusted for demographics and comorbidities.

**Results:**

The mean (SD) age of the cohort was 71 (10) years; the cohort was 96.7% (n = 13 828) male and 21.3% (n = 3043) African American, and 74.6% (n = 10 627) had diabetes. The 12-month all-cause mortality and CV mortality rates after 1 year of transition to dialysis were lower in statin continuers: deaths per 100 person-years were 21.9 (95% CI, 20.9-22.8) and 8.1 (95% CI, 7.5-8.6) in statin continuers vs 30.3 (95% CI, 27.8-32.8) and 9.8 (95% CI, 8.3-11.2) in statin discontinuers. Moreover, lower all-cause mortality and CV mortality risks with statin continuation persisted in adjusted analyses, with hazard ratios of 0.72 (95% CI, 0.66-0.79) and 0.82 (95% CI, 0.69-0.96), respectively. Associations were similar across subgroups, including age, race, and diabetes status.

**Conclusions and Relevance:**

In this study, the continuation of statin therapy after transition to dialysis was associated with reduced all-cause mortality and CV mortality. The study findings suggest that future studies are needed to examine potential CV benefits of continuing statin therapy after dialysis initiation.

## Key Points

**Question:**

Is the use of statins initiated during late-stage chronic kidney disease and continued after transition to dialysis associated with an improvement in subsequent survival?

**Findings:**

In this cohort study of 14 298 US veterans transitioning to dialysis, the continuation of statin therapy was associated with a 28% and 18% lower risk of 12-month all-cause mortality and cardiovascular mortality, respectively, compared with the discontinuation of statin therapy.

**Meaning:**

The continuation of statin therapy after transition to dialysis was associated with reduced all-cause mortality and cardiovascular mortality in veterans.

## Teaser

This cohort study compares whether the continuation vs discontinuation of statins from advanced chronic kidney disease into the dialysis therapy period is associated with improved all-cause mortality and cardiovascular mortality in US veterans.

## Introduction

Treatment of dyslipidemia in individuals with chronic kidney disease (CKD) is challenging. To reduce cardiovascular (CV) morbidity and mortality, current guidelines^1^ recommend lipid-lowering therapy, such as statins (hydroxymethylglutaryl coenzyme A reductase inhibitors), in patients with nondialysis-dependent CKD who are 50 years and older and in younger individuals with CKD and known coronary artery disease (CAD) or additional CAD risk factors. This recommendation is based on strong evidence from randomized clinical trials (RCTs) in patients with CKD, as well as subgroup analysis of patients with CKD from large RCTs conducted in unselected populations.^2,3,4,5,6^ However, de novo statin therapy has not been shown to be beneficial in patients with dialysis-dependent CKD in several well-conducted RCTs and thus is not advocated.^1,7,8,9^

Chronic kidney disease is highly prevalent and was found in 14.8% of individuals during 2011 through 2014 in the National Health and Nutrition Examination Survey.^10^ In 2014, close to 120 000 patients developed end-stage renal disease (ESRD) and transitioned to dialysis.^10^ According to Medicare Part D claims, statins top the list of the 15 most commonly prescribed drugs in patients with CKD, with reportedly 59% of patients receiving these medications.^10^ Therefore, a large proportion of patients with CKD are reaching ESRD while receiving statins. However, data from a recent United States Renal Data System (USRDS) chapter on US veterans transitioning to ESRD show that the proportion of patients receiving statin prescriptions decreased in the periods after ESRD transition compared with the periods before transition.^11^ It is unknown whether the cardioprotective effects of statins in nondialysis CKD extend into the ESRD period or, conversely, if the continuation of statins carries no CV benefit, as seen in de novo statin administration in individuals with ESRD. Even a neutral effect of statin use is potentially undesirable because patients receiving dialysis are already at the highest risk of polypharmacy and adverse effects from drug-drug interactions and altered drug metabolism. Moreover, there have been rising concerns that statin therapy may be directly harmful by promoting vascular calcification in patients with ESRD.^12,13,14^ The current expert opinion is to continue therapy with statins after transition to ESRD if this therapy with statins was started before dialysis initiation. However, the clinical evidence to support or refute this practice is lacking. Therefore, the objective of this study was to investigate whether the continuation of statin therapy from advanced CKD into the dialysis therapy period is associated with improved all-cause mortality and CV mortality in patients receiving dialysis.

## Methods

### Study Population and Data Source

We retrospectively examined data from the USRDS Transition of Care in Chronic Kidney Disease study,^11^ which focused on investigating patients transitioning to ESRD.^15,16^ In the present cohort study, we used the USRDS to identify the source cohort of 85 505 veterans who transitioned to ESRD between October 1, 2007, and March 30, 2014. Data analysis was conducted between August 2, 2017, and June 28, 2018. After excluding 1958 patients with missing censoring information, we excluded 25 792 patients who were missing prescription medication information in the year before ESRD transition, and we excluded 10 035 patients who received less than 6 months of statin prescription in the year before transition. We then excluded 17 469 patients with less than 1 year of follow-up after transition and 4827 patients with less than 6 months of statin prescription in the year after transition. Of 25 424 patients who met inclusion criteria, there were 14 298 patients who used statins in the year before transition to dialysis and 11 126 patients who did not use statins in the year before transition to dialysis. Our final main analytical cohort of 14 298 patients comprised 11 936 patients with continued statin use and 2362 patients with discontinued statin use after transition to dialysis (eFigure 1 in the Supplement).

The institutional review boards of the Tibor Rubin Veterans Affairs Medical Center and Memphis Veterans Affairs Medical Center approved this study, and the participant written informed consent requirement was waived given the large sample size, patient anonymity, and nonintrusive nature of the study. This study adhered to Strengthening the Reporting of Observational Studies in Epidemiology (STROBE) reporting guideline.

### Demographics and Clinical Measurements

Baseline characteristics, including age, sex, and self-identified race and ethnicity, were ascertained from the following 3 national databases: the USRDS, Veterans Affairs (VA), and Centers for Medicare & Medicaid Services (CMS). Data on marital status and smoking status at the time of transition to dialysis were obtained from VA records only.^17^ Data on cause of ESRD, initial dialysis modality, and initial access type were extracted from USRDS files. Preexisting comorbidities at transition were extracted from VA and CMS databases using *International Classification of Diseases*, *Ninth Revision* (*ICD-9*) diagnostic and procedural codes and *Current Procedural Terminology* codes as guided by the Deyo Charlson Comorbidity Index (CCI) and CMS chronic conditions. An algorithm for 1 inpatient visit or 2 outpatient visits was used to determine the presence of comorbidity. The CCI was calculated without renal disease. Cardiovascular disease (CVD) was defined as the presence of any prior ischemic heart disease, congestive heart failure, peripheral vascular disease, cerebrovascular disease, myocardial infarction, or atrial fibrillation. Moreover, occurrence of an acute kidney injury in the year before transition was obtained from hospitalization records with any admission diagnosis of *ICD-9* code 584.x.

Data on pre-ESRD (prelude) laboratory measurements were primarily sourced from VA databases. Serum creatinine level data and thus the last measurement of the estimated glomerular filtration rate within the past 90 days before transition were chiefly obtained from the VA Corporate Data Warehouse LabChem file and supplemented with the USRDS CMS 2728 Medical Evidence form. Serum albumin level at the time of transition was primarily obtained from the USRDS CMS 2728 file and was supplemented with data from the VA Corporate Data Warehouse LabChem file. Other serum laboratory measurements, including lipid panel, were obtained from the Decision Support System National Data Extracts Laboratory Results file. With the exception of estimated glomerular filtration rate and serum albumin level, laboratory measurements within the 1-year prelude period were averaged. Finally, we ascertained the number of prescribed medications at the time of transition and 6 months after transition from medication dispensation records.

### Exposure Measurement

Both inpatient and outpatient medication data were sourced from CMS Medicare Part D and VA pharmacy dispensation records.^18^ Lipid-lowering drugs, including statins, were extracted using specific drug-class codes or name. Patients covered for at least 6 months were characterized as receiving statins. Patients with any medication prescription in the year before or year after the dialysis period who were not prescribed a lipid-lowering drug in that period were categorized as not receiving statins. Our primary exposures were statin therapy continuation and statin treatment discontinuation, defined as receiving a statin before transition and either continuing or discontinuing statin prescription after transition to dialysis. In additional analyses, we also examined patients who were not receiving a statin before transition and either initiated use of statins or continued to not use statins after transition to dialysis (eFigure 1 in the Supplement). Newly initiated statin-receiving patients were covered by a statin prescription for at least 6 months of the year after transition to dialysis.

### Outcome Assessment

The primary and secondary outcomes were postdialysis all-cause mortality and CV mortality, respectively. Cardiovascular causes of death, including myocardial infarction, cardiac arrest, congestive heart failure, valvular heart disease, cardiac arrhythmia, cardiomyopathy, pericarditis, cerebrovascular event, pulmonary embolus, and atherosclerotic heart disease, were obtained from USRDS records only. All data on outcomes and censoring events were obtained from the USRDS, VA, and CMS data sets. Follow-up started 1 year after dialysis initiation. Our main follow-up period was 12-month mortality after 1 year of dialysis (ie, mortality between years 1 and 2 after transition). Patients were followed up until death, kidney transplant, loss to follow-up, or the end of the follow-up period or administrative censoring (June 30, 2014, for CV mortality or September 2, 2014, for all else), whichever occurred first.

### Statistical Analysis

Baseline patient characteristics are presented as the mean (SD) or median (interquartile range) for continuous variables and as proportions for categorical variables, as appropriate. Patients were compared between statin continuation groups with standardized differences.^19^

For the main analyses, the discontinued use of statin group was the referent group. The association of statin continuation with all-cause mortality and CV mortality was evaluated using Cox proportional hazards regression models. In secondary analyses, CV mortality was modeled using the Fine and Gray competing risk model, where non-CV mortality was considered a competing event.

Two models of hierarchical adjustment were used in each analysis. These were (1) an unadjusted model and (2) a model adjusted for demographics and comorbidities that included age, sex, race and ethnicity, and the following comorbidities: CCI, diabetes, atherosclerotic CV disease (defined as the presence of ischemic heart disease, peripheral vascular disease, or myocardial infarction), congestive heart failure, cerebrovascular disease, and atrial fibrillation.

We also examined the statin therapy continuation and mortality association with a priori selected subgroup analyses. Formal tests of interactions were performed using the Wald test under the adjusted model.

In additional analyses, we included patients who were not receiving statin therapy in both the pre-ESRD period and post-ESRD period and patients who initiated statin therapy after the transition to dialysis (statin initiators). Characteristics of these 4 statin use groups—none before or after ESRD, none before ESRD but statins after ESRD (statin initiators), statins before ESRD but none after ESRD (statin discontinuers), and statins before and after ESRD (statin continuers)—were compared using the χ^2^ test, analysis of variance, or Kruskal-Wallis test, where appropriate. Associations with mortality risks across these 4 groups were then examined using the none before or after ESRD patient group as the reference. Then, in sensitivity analyses, we revised the exposure definition and only removed patients who were censored within 42 days (6 weeks) after transition to dialysis (n = 1469) or did not have a prescription for any medication in the posttransition period (n = 1342). Patients who had an additional statin prescription within 42 days of the pretransition prescription end date (n = 13 635) or within 42 days of transition (n = 2313) were considered statin continuers (n = 15 948), whereas patients who had no statin prescription in the posttransition period (n = 2618) or exceeded 42 days to receive another statin prescription (n = 4192) were considered statin discontinuers (n = 6810) (eFigure 2 in the Supplement).

To further test the robustness of our findings in the original cohort, we also adjusted for the discontinuation of other medications, including absolute number of medication groups discontinued and specifically β-blockers, renin-angiotensin-aldosterone system inhibitors, and allopurinol. We also separately examined associations after additional adjustment for smoking, estimated glomerular filtration rate, lipid levels, number of prescribed medications at transition and 6 months after transition, initial access type, acute kidney injury diagnosis in the year before transition, and predialysis nephrology care in the VA and/or CMS in the year prior to transition. In addition, we conducted analyses using propensity score matching, adjustment, and stratification, as well as in a subset of patients after excluding those who withdrew from dialysis or had an unknown cause of death. Withdrawal from dialysis at least 1 year after transition was defined as dialysis discontinuation (identified from the USRDS treatment records) or as a listed cause of death. Furthermore, we also used restricted cubic splines to illustrate the association of the continuous number of days of statin use continuation after transition with all-cause mortality risk for patients who had at least 1 day of statin use in the posttransition year (n = 13 963, which includes the addition of 2027 patients with <6 months of statin treatment). Finally, we calculated E-values to ascertain the amount of bias in our estimated hazard ratios (HRs) due to uncontrolled confounding. E-values are a recently developed measurement used to determine the lowest strength of association needed between the confounder-exposure and confounder-outcome that would fully explain away our observed association between statin continuation and mortality.^20,21^

Data on demographics and comorbid conditions were missing for less than 0.4% of the cohort and imputed using a missingness category. Analyses were conducted using SAS Enterprise Guide 7.1 (SAS Institute Inc) or Stata 15 (StataCorp LP). *P* values were 2-sided, and *P* < .05 was considered significant.

## Results

The study cohort comprised 14 298 patients with pre-ESRD statin use who survived at least 1 year while receiving dialysis. The mean (SD) age of the cohort was 71 (10) years, the cohort was 96.7% (n = 13 828) male and 21.3% (n = 3043) African American, and 74.6% (n = 10 627) had diabetes (Table). In the cohort, 16.5% (n = 2362) discontinued statin use after transition to dialysis, whereas 83.5% (n = 11 936) continued to use statins in the first year after transition to dialysis. Across demographics and the presence of comorbidities, statin continuers and statin discontinuers were similar, including on the basis of presence of CV disease, age, and race and ethnicity. Statin continuers had a longer mean time receiving statins before transition to dialysis, had more prescribed prescriptions at both the time of transition and 6 months after transition, and had a higher proportion with an arteriovenous (AV) fistula or AV graft as their initial access type. They were also more likely to have nephrology visits, in particular as a VA outpatient in the year before transition.

**Table.** Baseline Characteristics of 14 298 Patients Stratified by Statin Continuation

Characteristic	Total	Statin Continuers	Statin Discontinuers	Standardized Difference^a^
Patients, No. (%)	14 298	11 936 (83.5)	2362 (16.5)	NA
Baseline cardiovascular disease, %				
No	16.6	16.6	17.1	0.01
Yes	83.4	83.5	82.9	
Ischemic heart disease	64.8	65.2	63.2	0.04
Congestive heart failure	56.1	56.0	56.5	−0.01
Peripheral vascular disease	40.6	40.7	40.3	0.01
Cerebrovascular disease	33.1	33.4	31.8	0.03
Myocardial infarction	27.7	27.8	27.1	0.02
Atrial fibrillation	14.8	14.8	15.1	−0.01
Time receiving statins, mean (SD), d				
Before transition to dialysis	280 (45)	282 (44)	269 (46)	0.31
After transition to dialysis	296 (50)	296 (50)	NA	NA
Age, mean (SD), y	71 (10)	71 (10)	71 (11)	−0.01
<65 y, %	29.0	29.2	28.1	0.02
65 to <75 y, %	29.4	29.4	29.4	0.00
≥75 y, %	41.6	41.4	42.5	−0.02
Male sex, %	96.7	96.8	96.4	−0.02
Race, %				
White	73.9	74.3	72.2	0.05
African American	21.3	20.8	23.5	−0.07
Other	4.8	4.9	4.3	0.03
Hispanic ethnicity, %	6.7	6.7	6.9	−0.01
Married, %	62.0	62.4	59.8	0.05
Deyo Charlson Comorbidity Index, median (IQR)	4 (2-6)	4 (2-5)	4 (2-6)	−0.05
Preexisting comorbidities, %				
Hyperlipidemia	90.5	90.6	89.8	0.03
Diabetes	74.6	74.8	73.7	0.03
Anemia	72.9	73.2	71.6	0.04
Chronic obstructive pulmonary disease	41.3	40.7	44.4	−0.07
Depression	22.6	22.4	22.0	−0.03
Cancer	21.8	21.7	22.6	−0.02
Liver disease	7.1	6.7	9.5	−0.10
Peptic ulcer disease	6.0	5.7	7.2	−0.06
Smoking status, %				
Never	30.2	30.0	31.3	0.04
Current	32.9	32.8	33.5	
Past	36.9	37.2	35.2	
Cause of end-stage renal disease, %				
Diabetes	51.5	52.1	48.6	0.07
Hypertension	28.9	28.7	29.9	
Glomerulonephritis	5.2	5.3	4.9	
Other/unknown	14.3	13.9	16.6	
eGFR at transition to dialysis, median (IQR), mL/min/1.73 m^2^	9.9 (7.4-13.1)	9.9 (7.5-13.0)	10.1 (7.3-13.6)	−0.10
Serum albumin at transition to dialysis, mean (SD), g/dL	3.3 (0.7)	3.4 (0.6)	3.3 (0.7)	0.16
12-mo averaged lipid levels, mg/dL				
HDL-C, mean (SD)	39 (13)	39 (13)	40 (14)	−0.10
LDL-C, mean (SD)	80 (31)	79 (30)	85 (36)	−0.19
Cholesterol, mean (SD)	149 (42)	148 (41)	155 (47)	−0.16
Triglycerides, median (IQR)	129 (91-186)	129 (91-187)	126 (89-185)	0.00
No. of prescribed medications, median (IQR)				
At the time of transition to dialysis	10 (6-16)	11 (7-17)	8 (5-12)	0.42
6 mo after transition to dialysis	8 (5-11)	8 (5-12)	4 (2-8)	0.68
Initial dialysis modality, %				
Hemodialysis	89.3	89.4	88.8	0.12
Peritoneal dialysis	6.4	6.6	5.3	
Other/unknown	4.3	4.0	5.9	
Initial access type, %				
AV fistula/AV graft	28.3	29.6	21.5	0.21
Central venous catheter	63.5	61.9	71.8	
Other/unknown	8.2	8.6	6.7	
AKI diagnosis in the year before transition to dialysis, %	24.4	23.1	31.2	−0.18

Abbreviations: AKI, acute kidney injury; AV, arteriovenous; eGFR, estimated glomerular filtration rate; HDL-C, high-density lipoprotein cholesterol; IQR, interquartile range; LDL-C, low-density lipoprotein cholesterol; NA, not applicable.

SI conversion factors: To convert albumin level to grams per liter, multiply by 10; to convert cholesterol level to millimoles per liter, multiply by 0.0259; to convert triglycerides level to millimoles per liter, multiply by 0.0113.

^a^ Data are compared between groups using standardized differences. Standardized differences of at least 0.2 are considered a meaningful imbalance, where 0.8, 0.5, and 0.2 represent large, medium, and small imbalances, respectively.

### Association of Statin Therapy Continuation With All-Cause Mortality and CV Mortality

**Figure 1.**
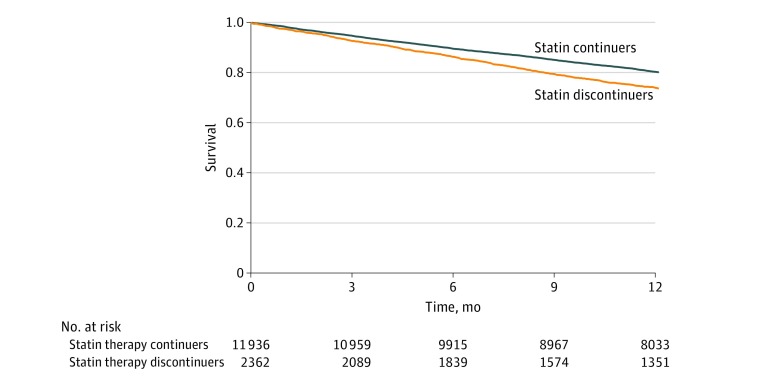
Kaplan-Meier Survival Plot Survival differences are shown in statin therapy continuers and statin therapy discontinuers in the second year after dialysis initiation.

**Figure 2.**
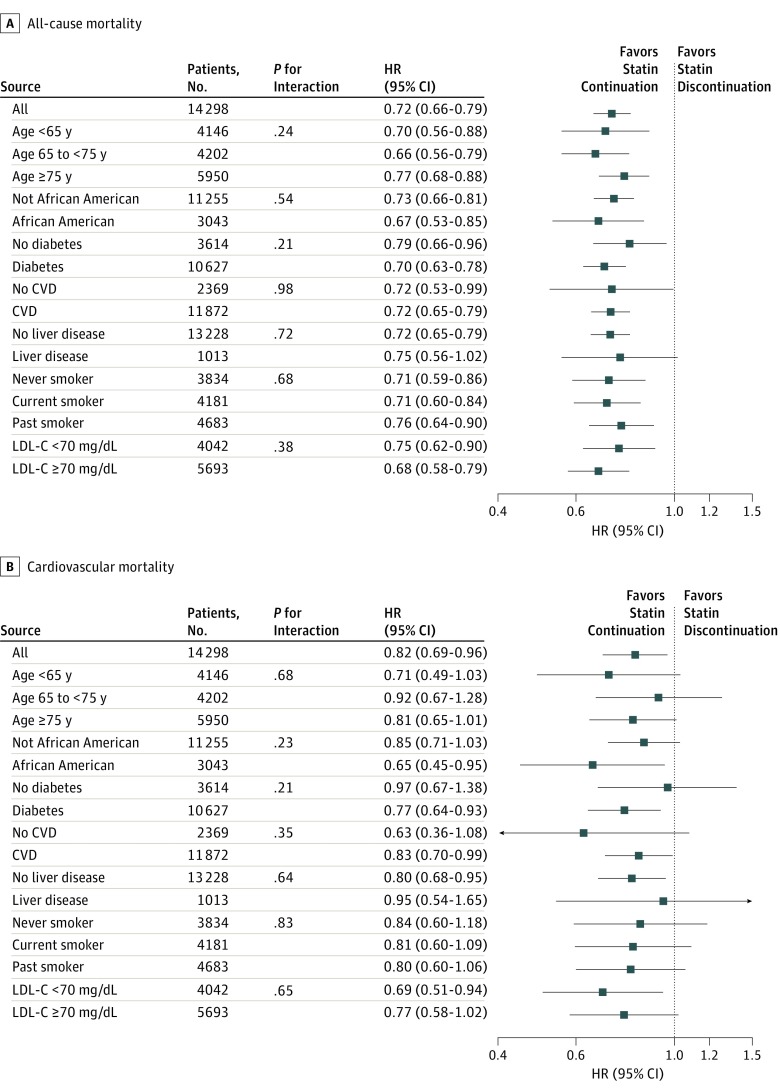
Association of Statin Therapy Continuation With All-Cause Mortality and Cardiovascular Mortality Association of statin therapy continuation with 12-month all-cause mortality (A) and 12-month cardiovascular mortality (B) is shown across a priori subgroups. The discontinued statin group was the referent group. Adjusted for age, sex, race, ethnicity, Deyo Charlson Comorbidity Index, and presence of diabetes, atherosclerotic cardiovascular disease (defined as presence of myocardial infarction, peripheral vascular disease, or ischemic heart disease), atrial fibrillation, congestive heart failure, and cerebrovascular disease. CVD indicates cardiovascular disease; HR, hazard ratio; and LDL-C, low-density lipoprotein cholesterol. To convert LDL-C level to millimoles per liter, multiply by 0.0259.

In the 12 months of follow-up after a year of dialysis, 2740 patients (19.2%) died, 169 (1.2%) were censored for kidney transplant, and 353 (2.5%) were lost to follow-up. Patients who continued statins had a lower crude all-cause mortality rate compared with patients who discontinued statins (21.9; 95% CI, 20.9-22.8 vs 30.3; 95% CI, 27.8-32.8 deaths per 100 person-years). This difference in crude mortality rate was evident in Kaplan-Meier analyses (Figure 1). In both unadjusted and adjusted models, patients who continued use of statins through transition to dialysis had a 28% lower risk of 12-month all-cause mortality compared with patients who discontinued use of statins (HR, 0.72; 95% CI, 0.66-0.79 for both models) (eTable 1 in the Supplement). We further examined the statin continuation and 12-month mortality association across a priori selected subgroups. Across all strata of demographics and comorbidities, statin continuation was associated with a lower risk of 12-month all-cause mortality (Figure 2A). There were no significant interactions for these associations.

Similar to all-cause mortality, statin continuers had a lower crude rate of 12-month CV mortality compared with statin discontinuers (8.1; 95% CI, 7.5-8.6 vs 9.8; 95% CI, 8.3-11.2 CV deaths per 100 person-years). Moreover, we observed a lower risk of 12-month CV mortality among statin continuers compared with statin discontinuers in adjusted analyses (fully adjusted HR, 0.82; 95% CI, 0.69-0.96 [eTable 2 in the Supplement]). In sensitivity analyses using a competing risk model, statin use continuation was associated with a lower risk of 12-month CV mortality, albeit slightly attenuated (adjusted subdistribution hazard ratio, 0.86; 95% CI, 0.73-1.01). Similar to all-cause mortality, statin continuation across a priori selected subgroups was largely associated with a lower risk of CV mortality, although these associations were strongly attenuated (Figure 2B). There were no significant interactions in the statin continuation and CV mortality association.

### Sensitivity Analyses

In sensitivity analyses, we attempted to minimize potential survival and selection biases by revising the cohort and the exposure definition to only exclude patients who were censored within 42 days after transition to dialysis and did not have prescription data in the posttransition period. Results of the sensitivity analyses similarly showed that patients continuing use of statins had a lower risk of all-cause mortality and CV mortality (eTable 3 in the Supplement). Moreover, among 13 963 patients with at least 1 day of statin treatment in the post-ESRD period, we observe a gradual and inverse association between number of days receiving statins and all-cause mortality. Patients with a longer duration of statin use had a lower risk of all-cause mortality (eFigure 3 in the Supplement).

**Figure 3.**
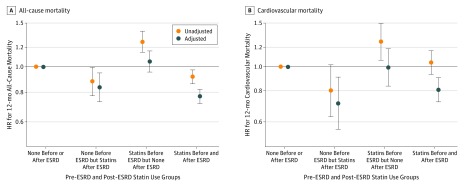
Association of Statin Use With All-Cause Mortality and Cardiovascular Mortality Association of statin use before and/or after end-stage renal disease (ESRD) transition with 12-month all-cause mortality (A) and 12-month cardiovascular mortality (B) among 25 424 patients is shown. Error bars represent 95% CIs for the hazard ratios (HRs). Models were unadjusted or adjusted for age, sex, race, ethnicity, Deyo Charlson Comorbidity Index, and presence of diabetes, atherosclerotic cardiovascular disease (defined as presence of myocardial infarction, peripheral vascular disease, or ischemic heart disease), atrial fibrillation, congestive heart failure, and cerebrovascular disease.

In additional analyses, among the 25 424 patients who survived the first year of dialysis, we compared patients who were not prescribed a statin in both the predialysis and postdialysis 1-year periods with patients who were initiators of statin therapy after dialysis, statin continuers, and statin discontinuers. We observed that 38.1% of the cohort were not prescribed statins in both the predialysis and postdialysis periods, whereas 5.6% of patients newly initiated use of statin after transition to dialysis. Both of these patient groups were more likely to be younger and of African American race and in general had less comorbidity, including CVD, diabetes, and anemia. In addition, patients who were not prescribed statins in either period were more likely to have liver disease (eTable 4 in the Supplement). Patients who initiated statin treatment after dialysis were less likely to have a nephrology visit in the year before transition or to have an AV fistula or AV graft as their access type at dialysis initiation. Compared with patients who were not prescribed statins in both the predialysis and postdialysis periods, those who continued statins still had the lowest risk of 12-month all-cause mortality after adjustment for demographics and comorbidities. Patients who newly initiated statin therapy after dialysis likewise had a lower risk of mortality (Figure 3 and eTable 5 in the Supplement). This association was similar for CV mortality, where patients who used statins after transition to dialysis (either newly initiated or continued) had a lower risk of 12-month mortality compared with patients who did not use statins. In contrast, patients who discontinued use of statins had no difference in risk compared with patients who were not prescribed statins before and after dialysis initiation (Figure 3 and eTable 5 in the Supplement).

In sensitivity analyses, associations of statin continuation with all-cause mortality risk persisted after adjustment for several additional covariates (eTable 6 in the Supplement) and propensity score matching (eTable 7 in the Supplement). For the statin continuation and all-cause mortality association, the estimated E-value was 1.82, with a lower confidence limit of 1.63. Statin continuers were more likely to continue other selected medications (eTable 8 and eTable 9 in the Supplement); however, after adjustment for the continuation or discontinuation of treatment with these other medications, associations remained the same. There were 1340 patients, including 1108 statin continuers and 232 statin discontinuers, who withdrew from dialysis in the second year after transition. No significant difference in the percentage of withdrawal patients was observed between groups . After removing these patients, statin continuation continued to be associated with a lower mortality risk in adjusted models (HR, 0.76; 95% CI, 0.68-0.85). After also removing patients who had an unknown cause of death (n = 1865), statin continuation was similarly associated with a lower risk of CV mortality as in the primary analytical cohort (HR, 0.80; 95% CI, 0.68-0.94) but not with non-CV mortality in adjusted models (HR, 0.98; 95% CI, 0.76-1.27).

## Discussion

This retrospective cohort study demonstrated that the continuation of statin therapy initiated in predialysis CKD into the post-ESRD period was associated with lower all-cause mortality and CV mortality after the transition to dialysis. Patients with CKD who continued therapy with statins for at least 6 months during the first year after the transition to dialysis had a 28% lower risk of death and 18% lower risk of fatal CV events during the subsequent 12 months compared with patients who discontinued statin therapy. This benefit did not differ across subgroups of demographics and clinical characteristics. There was no significant difference in benefit between patients who achieved a 1-year averaged pretransition serum low-density lipoprotein cholesterol level lower than 70 mg/dL compared with those who did not achieve this degree of serum low-density lipoprotein cholesterol level reduction (to convert cholesterol level to millimoles per liter, multiply by 0.0259). As expected, the CV mortality data paralleled the all-cause mortality data, showing a significant benefit and no heterogeneity between subgroups.

Three RCTs failed to show benefit from initiation of statin therapy in patients receiving hemodialysis^2,7,8^ (eTable 10 in the Supplement). Current guidelines^22,23,24^ and evidence-based reviews^9,25,26,27^ are mainly focused on results of these studies. The guidelines recommend that statins should not be initiated in patients undergoing dialysis but should be continued in patients receiving statins before dialysis. The latter recommendation was based on a subgroup analysis of patients who had nondialysis-dependent–CKD (NDD-CKD) at baseline from the Study of Heart and Renal Protection (SHARP).^2^ Among this group, 34% of the 6247 patients transitioned to dialysis over the 4.9 years of follow-up. The patients with NDD-CKD who transitioned to dialysis were not analyzed separately from those who did not. The trial demonstrated that in patients with NDD-CKD, the combination of simvastatin and ezetimibe vs placebo resulted in a 22% reduction of major CV events, consisting of nonfatal myocardial infarction, coronary death, nonhemorrhagic stroke, or any arterial revascularization procedure, and this benefit was interpreted as applicable to all patients with NDD-CKD. However, it was still unknown if patient outcomes would differ between patients with NDD-CKD who continue statin therapy while transitioning to dialysis and those who do not transition. Furthermore, these findings did not address the key question of whether statin therapy should be continued in patients transitioning to dialysis.

Results of prior studies have also suggested concerns about the use of statin therapy in patients receiving dialysis. An analysis by the Cholesterol Treatment Trialists’ Collaboration^3^ showed a significant trend in decreasing statin effectiveness with progression of CKD, presumably attributable to increasing incidence of fatal and nonfatal events related to nonatherosclerotic CVD. Other authors have suggested that statins are less efficient in patients who are high cholesterol absorbers,^28,29^ and some researchers have noted that patients receiving dialysis have increased cholesterol absorption.^30^ Moreover, there is evidence^12,31^ that statins can increase vascular calcification as a part of the plaque stabilization process^14,32^ and that vascular calcification is a strong risk factor for mortality in patients receiving dialysis.^33^ Given these compelling findings, some authors have even called for a moratorium on statin use in patients receiving dialysis^13^ and have proposed statin therapy discontinuation while transitioning to dialysis.^34^

In our cohort, initiating use of statins after transition to dialysis was associated with a lower subsequent mortality risk. This finding may be attributed to an improvement in care after a patient transitions to dialysis. Treatment with statins is indicated for all patients with late-stage CKD according to current guidelines.^1^ In our study, these patients with CKD did not receive statins in the year before transition; furthermore, they were less likely to have nephrology outpatient visits in the year before transition or have an AV fistula or AV graft as their access type at the time of dialysis initiation (an additional proxy of predialysis care). However, our study results are supported by findings of most previous cohort studies examining the association of statin therapy with outcomes in patients receiving dialysis. Early data from the USRDS Morbidity and Mortality Wave-2 study,^35^ including 3716 patients initiating dialysis in 1996, showed that statin use was associated with lower all-cause mortality and CV mortality for up to 1.5 years of follow-up. Similar results were reported from the Dialysis Outcomes and Practice Patterns Study^36^ and in the Taiwan National Health Insurance Research Database study.^37^ Other studies have found similar associations for specific dialysis populations, including those who sustained a myocardial infarction,^38,39,40^ those who underwent vascular access placement surgery,^41^ and those who had diabetes.^42,43^ In addition, a meta-analysis^44^ that included both RCTs and cohort studies showed that the effect of statin use in patients with diabetes receiving dialysis was associated with a lower risk of all combined cardiac events and all-cause mortality.

Our study is clinically important because it provides support for the guidelines^1^ recommending the continuation of statin therapy in patients transitioning to dialysis. It is notable for its large size, and to date it is the first to explore whether the continuation of statin therapy is associated with improved survival among patients transitioning from the predialysis to postdialysis periods. Moreover, given the use of both the VA and CMS databases, we had data on prescriptions both before and after transition. Because current guidelines advocate for the continuation of statins during dialysis, an observational study design may be necessary as an RCT to study statin discontinuation may be deemed unethical.

### Limitations

Several limitations of the present analysis should be considered. This was an observational study that included predominantly male veterans; therefore, despite careful adjustment for baseline differences between statin continuers and discontinuers and the performance of several sensitivity analyses, the presence of residual biases affecting all-cause mortality and CV mortality cannot be excluded. In particular, residual confounding is possible; however, for an unmeasured characteristic to render the described association between statin continuation and mortality nonsignificant, it would have to show an adjusted risk ratio of at least 1.82 (or 1.63 to explain away the lower confidence limit) with both the exposure and the outcome separately. While we cannot rule out the presence of such an unmeasured confounder, of the measured patient characteristics included in our multivariable model, no covariate satisfied this requirement (eTable 11 in the Supplement).

In addition, the reasons why statin treatments were stopped after dialysis initiation are unknown and could have led to a selection bias and/or confounding by indication. There also may be problems of treatment adherence or discontinuation due to polypharmacy, adverse effects, or noneffectiveness. Furthermore, we cannot exclude use of statins were discontinued in patients with expected limited life span, which in turn could result in lower survival in these patients independent of the effect of statin treatment cessation. However, to control for this confounding, we excluded patients with early mortality (during the first year after initiation of dialysis), and the benefit of statin continuation was still present, as well as in subgroups of patients with baseline CVD, age 75 years or older, and diabetes.

## Conclusions

Extension of statin therapy after transition to dialysis was associated with reduced all-cause mortality and CV mortality. However, until additional clinical data are available, it is critical to weigh potential CV benefits of continuing use of statins after dialysis initiation.

## Notes

Supplement. **eFigure 1.** Cohort Construction for Four Group Additional Analysis **eFigure 2.** Cohort Construction for Sensitivity Analysis, Revised Exposure and Cohort Definition **eFigure 3.** Restrictive Cubic Spline Illustrating Association of Days Continued on Statin in the Year Post-Transition With All-Cause Mortality Risk in the Subsequent Year in 13963 Patients with at Least One Day of Statin Treatment in the Year Post-Transition **eTable 1.** Event Rate and Hazard Ratios for the Association of Statin Continuation (ref: Statin Discontinuation) With All-Cause Mortality Over 12-Months of Follow-up **eTable 2.** Event Rate and Hazard Ratios for the Association of Statin Continuation (ref: Statin Discontinuation) With Cardiovascular Mortality Over 12-Months of Follow-up **eTable 3.** Event Rate and Hazard Ratios for the Association of Statin Continuation (ref: Statin Discontinuation) With A) All-Cause Mortality and B) Cardiovascular Mortality Over 12-Months of Follow-up in 22,758 Patients With Revised Exposure Definition **eTable 4.** Baseline Characteristics across 4 Statin Use Groups in the Pre and Post ESRD period in 25,424 Patients **eTable 5.** Event Rate and Hazard Ratios for the Association of Statin Pre and Post Transition Groups (ref: None Before or After ESRD) With A) All-Cause Mortality and B) Cardiovascular Mortality Over 12-Months of Follow-up **eTable 6.** Sensitivity Analysis Showing Associations of Statin Continuation vs. Discontinuation With All-Cause Mortality Risk After Additional Adjustment for Other Factors **eTable 7.** Sensitivity Analysis Showing Associations of Statin Continuation vs. Discontinuation With All-Cause Mortality Risk After Propensity Score Matching, Adjustment, and Stratification **eTable 8.** Medication Groups Ever Prescribed in the Year Pre and Post Transition to Dialysis in Statin Continuation and Statin Discontinuation Patients **eTable 9.** Discontinuation of Other Medications **eTable 10.** Studies Investigating Statin Use in Dialysis Patients **eTable 11.** Risk Ratios for Statin Continuation, Hazard Ratios for All-Cause Mortality, and Estimate E-Values for Covariates Included in Fully Adjusted Model
