# Supplementary material for: Association of Continuation of Statin Therapy Initiated Before Transition to Chronic Dialysis Therapy With Mortality After Dialysis Initiation
Source: JAMA Netw Open. 2018 Oct 5;1(6):e182311. doi: 10.1001/jamanetworkopen.2018.2311 (PMC6324660; doi:10.1001/jamanetworkopen.2018.2311)
Supplement: Supplement. — eFigure 1. Cohort Construction for Four Group Additional Analysis eFigure 2. Cohort Construction for Sensitivity Analysis, Revised Exposure and Cohort Definition eFigure 3. Restrictive Cubic Spline Illustrating Association of Days Continued on Statin in the Year Post-Transition With All-Cause Mortality Risk in the Subsequent Year in 13963 Patients with at Least One Day of Statin Treatment in the Year Post-Transition eTable 1. Event Rate and Hazard Ratios for the Association of Statin Continuation (ref: Statin Discontinuation) With All-Cause Mortality Over 12-Months of Follow-up eTable 2. Event Rate and Hazard Ratios for the Association of Statin Continuation (ref: Statin Discontinuation) With Cardiovascular Mortality Over 12-Months of Follow-up eTable 3. Event Rate and Hazard Ratios for the Association of Statin Continuation (ref: Statin Discontinuation) With A) All-Cause Mortality and B) Cardiovascular Mortality Over 12-Months of Follow-up in 22,758 Patients With Revised Exposure Definition eTable 4. Baseline Characteristics across 4 Statin Use Groups in the Pre and Post ESRD period in 25,424 Patients eTable 5. Event Rate and Hazard Ratios for the Association of Statin Pre and Post Transition Groups (ref: None Before or After ESRD) With A) All-Cause Mortality and B) Cardiovascular Mortality Over 12-Months of Follow-up eTable 6. Sensitivity Analysis Showing Associations of Statin Continuation vs. Discontinuation With All-Cause Mortality Risk After Additional Adjustment for Other Factors eTable 7. Sensitivity Analysis Showing Associations of Statin Continuation vs. Discontinuation With All-Cause Mortality Risk After Propensity Score Matching, Adjustment, and Stratification eTable 8. Medication Groups Ever Prescribed in the Year Pre and Post Transition to Dialysis in Statin Continuation and Statin Discontinuation Patients eTable 9. Discontinuation of Other Medications eTable 10. Studies Investigating Statin Use in Dialysis Patients eTable 11. Risk Ratios for Statin Co [file jamanetwopen-1-e182311-s001.pdf]

Supplementary Online Content

Streja E, Gosmanova EO, Molnar MZ, et al. Association of continuation of statin therapy initiated before transition to chronic dialysis therapy with mortality after dialysis initiation. *JAMA Netw Open*. 2018;1(6):e182311. doi:10.1001/jamanetworkopen.2018.2311

eFigure 1. Cohort Construction for Four Group Additional Analysis

eFigure 2. Cohort Construction for Sensitivity Analysis, Revised Exposure and Cohort Definition

eFigure 3. Restrictive Cubic Spline Illustrating Association of Days Continued on Statin in the Year Post-Transition with All-Cause Mortality Risk in the Subsequent Year in 13963 Patients With at Least One Day of Statin Treatment in the Year Post-Transition

eTable 1. Event Rate and Hazard Ratios for the Association of Statin Continuation (ref: Statin Discontinuation) With All-Cause Mortality Over 12-Months of Follow-up

eTable 2. Event Rate and Hazard Ratios for the Association of Statin Continuation (ref: Statin Discontinuation) With Cardiovascular Mortality Over 12-Months of Follow-up

eTable 3. Event Rate and Hazard Ratios for the Association of Statin Continuation (ref: Statin Discontinuation) With A) All-Cause Mortality and B) Cardiovascular Mortality Over 12-Months of Follow-up in 22,758 Patients With Revised Exposure Definition

eTable 4. Baseline Characteristics across 4 Statin Use Groups in the Pre and Post ESRD period in 25,424 Patients

eTable 5. Event Rate and Hazard Ratios for the Association of Statin Pre and Post Transition Groups (ref: None Before or After ESRD) With A) All-Cause Mortality and B) Cardiovascular Mortality Over 12-Months of Follow-up

eTable 6. Sensitivity Analysis Showing Associations of Statin Continuation vs. Discontinuation With All-Cause Mortality Risk After Additional Adjustment for Other Factors

eTable 7. Sensitivity Analysis Showing Associations of Statin Continuation vs. Discontinuation With All-Cause Mortality Risk After Propensity Score Matching, Adjustment, and Stratification

eTable 8. Medication Groups Ever Prescribed in the Year Pre and Post Transition to Dialysis in Statin Continuation and Statin Discontinuation Patients

eTable 9. Discontinuation of Other Medications

eTable 10. Studies Investigating Statin Use in Dialysis Patients

© 2018 Streja E et al. *JAMA Network Open.*

eTable 11. Risk Ratios for Statin Continuation, Hazard Ratios for All-Cause Mortality, and Estimate E-Values for Covariates Included in Fully Adjusted Model

This supplementary material has been provided by the authors to give readers additional information about their work.

© 2018 Streja E et al. *JAMA Network Open.*

eFigure 1. Cohort Construction for Four Group Additional Analysis

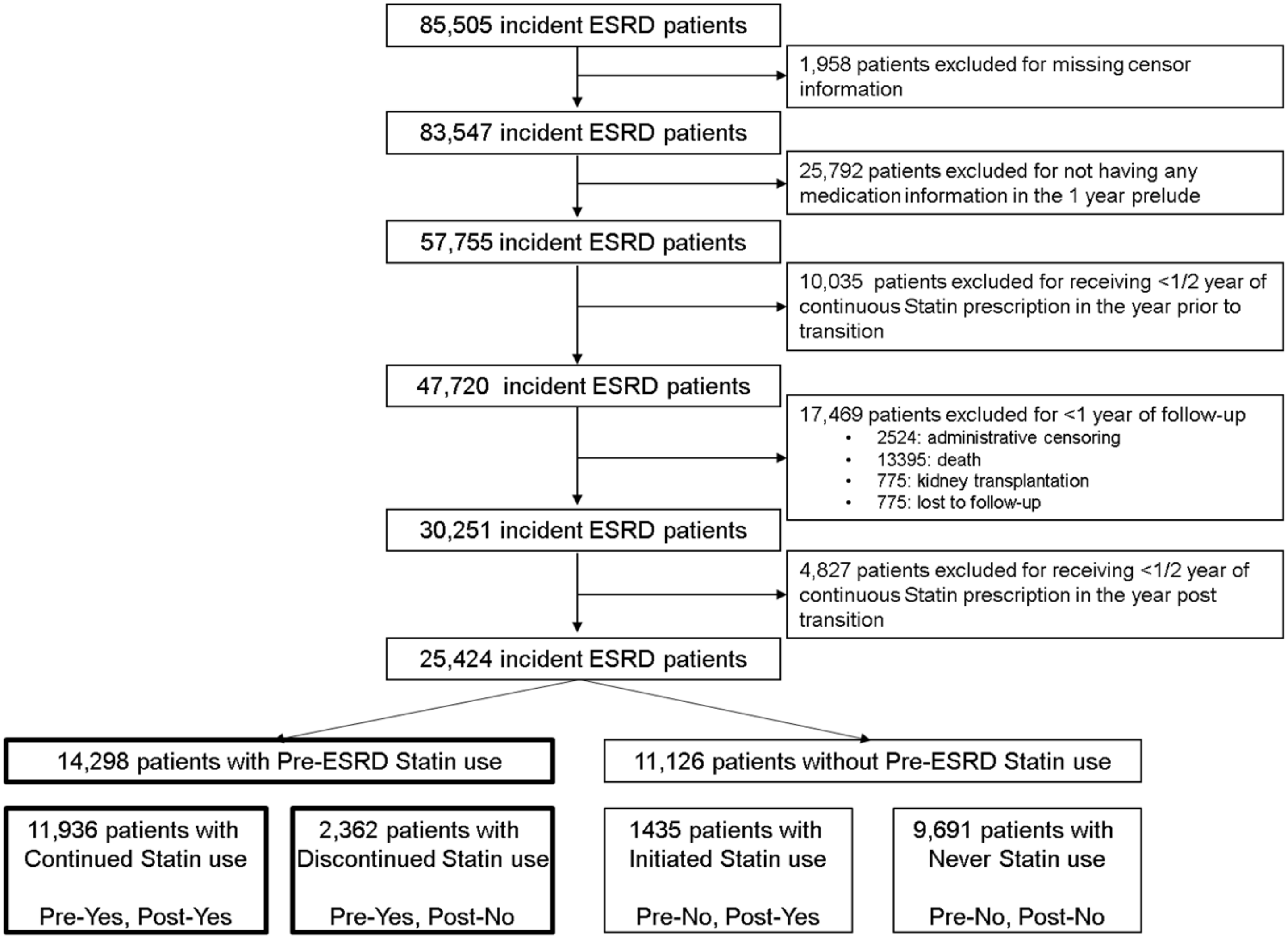

© 2018 Streja E et al. *JAMA Network Open.*

eFigure 2. Cohort Construction for Sensitivity Analysis, Revised Exposure and Cohort Definition

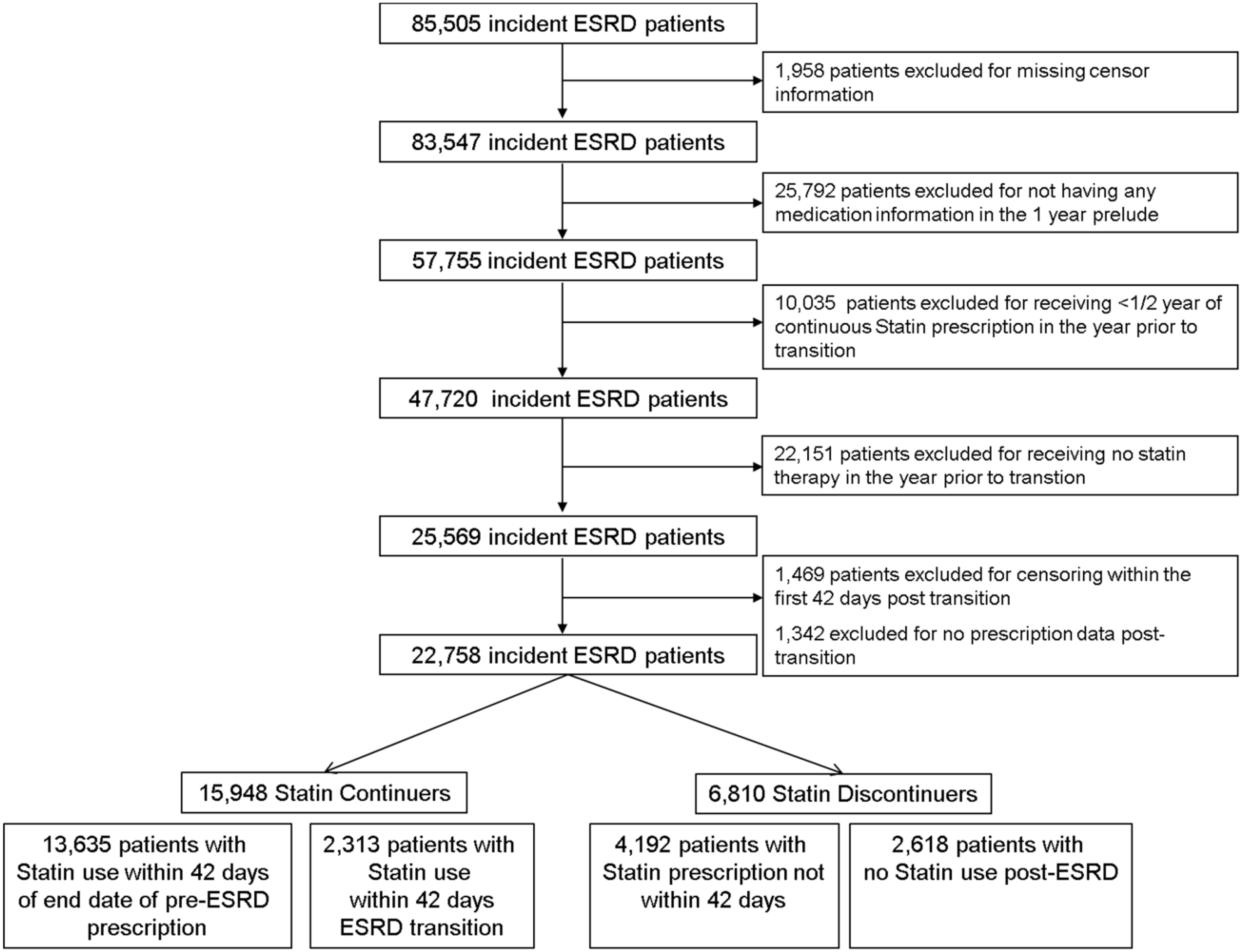

© 2018 Streja E et al. *JAMA Network Open.*

eFigure 3. Restrictive Cubic Spline Illustrating Association of Days Continued on Statin in the Year Post-Transition with All-Cause Mortality Risk in the Subsequent Year in 13963 Patients With at Least One Day of Statin Treatment in the Year Post-Transition

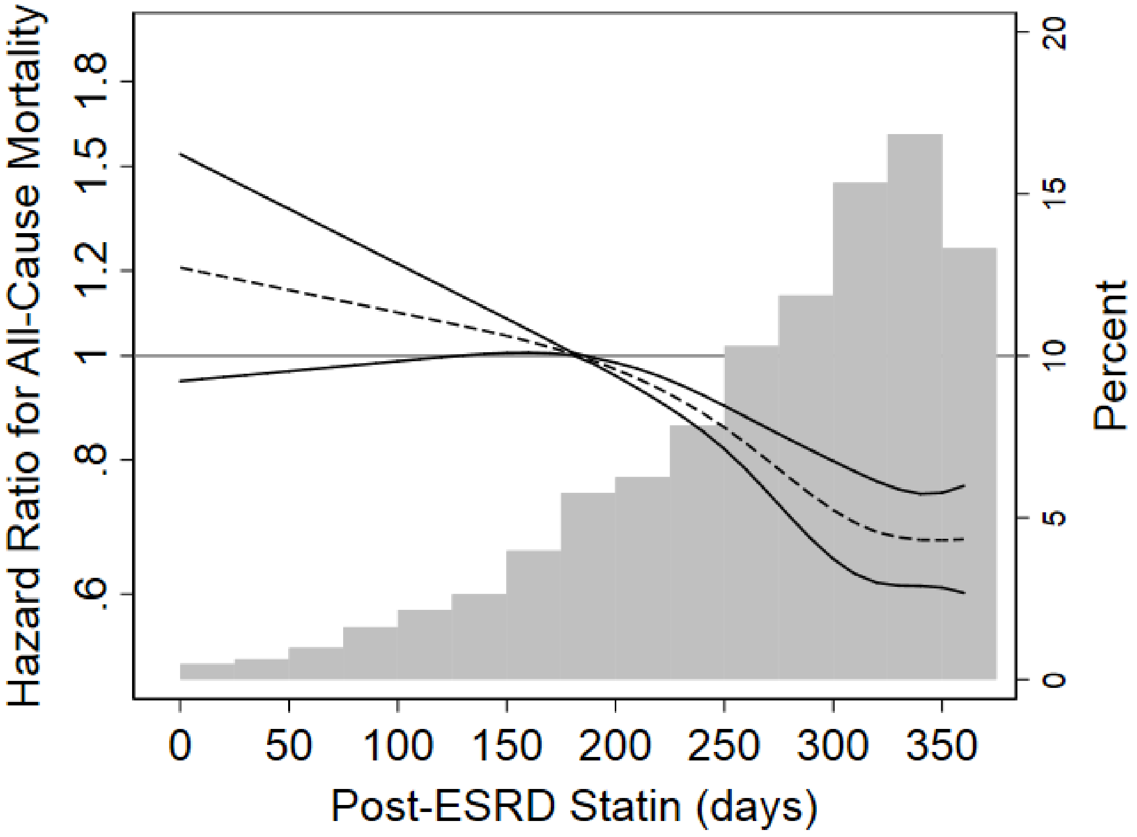

Adjusted covariates: age, gender, race, and ethnicity as well as the following comorbidities: CCI, diabetes, ASCVD (defined as presence of MI, PVD, or ISHD), atrial fibrillation, CHF and cerebrovascular disease.

© 2018 Streja E et al. *JAMA Network Open.*

eTable 1. Event Rate and Hazard Ratios for the Association of Statin Continuation (ref: Statin Discontinuation) With All-Cause Mortality Over 12-Months of Follow-up

All-Cause Mortality						
Group	Events	Crude Rate per 100 person-years [95%CI]	Unadjusted		Adjusted	
			HR[95%CI]	p-value	HR[95%CI]	p-value
**12-month**						
Total (n=14,298)	2740 (19%)	23.2[22.3,24.0]				
Discontinued (n=2,362)	558 (24%)	30.3[27.8,32.8]	Ref		Ref	
Continued (n=11,936)	2182 (18%)	21.9[20.9,22.8]	0.72[0.66,0.79]	<.001	0.72[0.66,0.79]	<.001

Adjusted covariates: age, gender, race, and ethnicity as well as the following comorbidities: CCI, diabetes, ASCVD (defined as presence of MI, PVD, or ISHD), atrial fibrillation, CHF and cerebrovascular disease.

© 2018 Streja E et al. *JAMA Network Open.*

eTable 2. Event Rate and Hazard Ratios for the Association of Statin Continuation (ref: Statin Discontinuation) With Cardiovascular Mortality Over 12-Months of Follow-up

Cardiovascular Mortality										
Group	Events	Crude Rate per 100 person-years [95%CI]	**Cox**				**Competing Risk**			
			Unadjusted		Adjusted		Unadjusted		Adjusted	
			HR[95%CI]	p-value	HR[95%CI]	p-value	HR[95%CI]	p-value	HR[95%CI]	p-value
**12-month**										
Total (n=14,298)	958 (7%)	8.3 [7.8,8.9]								
Discontinued (n=2,362)	174 (7%)	9.8 [8.3,11.2]	Ref		Ref		Ref		Ref	
Continued (n=11,936)	784 (7%)	8.1 [7.5,8.6]	0.82 [0.70,0.97]	0.02	0.82 [0.69,0.96]	0.02	0.85 [0.72,1.01]	0.06	0.86 [0.73,1.01]	0.06

Adjusted covariates: age, gender, race, and ethnicity as well as the following comorbidities: CCI, diabetes, ASCVD (defined as presence of MI, PVD, or ISHD), atrial fibrillation, CHF and cerebrovascular disease.

© 2018 Streja E et al. *JAMA Network Open.*

eTable 3. Event Rate and Hazard Ratios for the Association of Statin Continuation (ref: Statin Discontinuation) With A) All-Cause Mortality and B) Cardiovascular Mortality Over 12-Months of Follow-up in 22,758 Patients With Revised Exposure Definition

A)

**All-Cause Mortality**						
Group	Events	Crude Rate per 100 person-years [95%CI]	Unadjusted		Adjusted	
			HR[95%CI]	p-value	HR[95%CI]	p-value
**12-month**						
Total (n=22758)	4862 (21%)	28.1[27.3,28.9]				
Discontinued (n=6810)	1870 (27%)	37.6[35.9,39.4]	Ref		Ref	
Continued (n=15948)	2992 (19%)	24.2[23.3,25.1]	0.64[0.61,0.68]	<.001	0.71[0.67,0.75]	<.001

B)

**Cardiovascular Mortality**										
Group	Events	Crude Rate per 100 person-years [95%CI]	**Cox**				**Competing Risk**			
			Unadjusted		Adjusted		Unadjusted		Adjusted	
			HR [95%CI]	p-value	HR [95%CI]	p-value	HR [95%CI]	p-value	HR [95%CI]	p-value
**12-month**										
Total (n=22758)	1633 (7%)	9.2 [8.7,9.6]								
Discontinued (n=6810)	609 (9%)	11.8 [10.8,12.7]	Ref		Ref		Ref		Ref	
Continued (n=15948)	1024 (6%)	8.1 [7.6,8.6]	0.69 [0.63,0.76]	<.001	0.76 [0.68,0.84]	<.001	0.72 [0.66,0.80]	<.001	0.79 [0.72,0.88]	<.001

Adjusted covariates: age, gender, race, and ethnicity as well as the following comorbidities: CCI, diabetes, ASCVD (defined as presence of MI, PVD, or ISHD), atrial fibrillation, CHF and cerebrovascular disease.

© 2018 Streja E et al. *JAMA Network Open.*

eTable 4. Baseline Characteristics across 4 Statin Use Groups in the Pre and Post ESRD period in 25,424 Patients

		Statin Pre and Post Transition Groups				
	Total	None Before or After ESRD	None Before ESRD but Statins After ESRD	Statins Before ESRD but None After	Statins Before and After ESRD	P-value
N, (%)	25424	9691 (38.1)	1435 (5.6)	2362 (9.3)	11936 (46.9)	
Cardiovascular Disease (%)						<.001
No	25	37	26	17	17	
Yes	75	63	74	83	83	
ISHD	55	40	55	63	65	<.001
CHF	50	42	50	56	56	<.001
PVD	35	28	31	40	41	<.001
Cerebrovascular Disease	28	21	26	32	33	<.001
MI	23	15	25	27	28	<.001
Atrial Fibrillation	14	12	10	15	15	<.001
Time on statin prior to initiation (days)	280±45	N/A	N/A	269±46	282±44	<.001
Time on statin after initiation (days)	292±51	N/A	263±47	N/A	296±50	<.001
Age (years)	70±11	69±12	69±11	71±11	71±10	<.001
Age <65 (%)	33	39	36	28	29	<.001
Age 65-<75 (%)	27	24	27	29	29	<.001
Age≥75 (%)	40	37	37	43	41	<.001
Gender (%)						<.001
Female	4	5	4	4	3	
Race (%)						
White	70	64	70	72	74	<.001
African-American	25	31	25	24	21	<.001
Other	5	5	5	4	5	0.41
Ethnicity (%)						0.07
Hispanic	7	7	8	7	7	
Married Status (%)	59	55	60	60	62	<.001
CCI	4[2,5]	3[1,5]	3[2,5]	4[2,6]	4[2,5]	<.001
Preexisting comorbidities (%)						
Hyperlipidemia	77	57	71	90	91	<.001
Diabetes	66	55	64	74	75	<.001
Anemia	70	67	64	72	73	<.001
COPD	38	35	33	44	41	<.001
Depression	22	22	17	24	22	<.001
Cancer	22	23	18	23	22	<.001
Liver Disease	10	15	8	9	7	<.001
Peptic Ulcer	6	6	6	7	6	0.05

© 2018 Streja E et al. *JAMA Network Open.*

Disease						
Smoking Status (%)						<.001
Never	30	30	31	31	30	
Current	35	39	36	34	33	
Past	34	31	33	35	37	
Cause of ESRD(%)						<.001
Diabetes	45	35	48	49	52	
Hypertension	32	36	32	30	29	
Glomerulonephritis	6	8	6	5	5	
Other/Unknown	17	22	15	17	14	
eGFR at transition to dialysis, (mL/min/1.73 m^2)	9.6 [7.1,13.0]	9.2 [6.6,12.6]	9.5 [6.9,13.2]	10.1 [7.3,13.6]	9.9 [7.5,13.0]	<.001
Serum albumin at transition to dialysis, (g/dL)	3.3±0.7	3.3±0.7	3.3±0.7	3.3±0.7	3.4±0.6	<.001
12-month Averaged Lipids, mg/dL						
HDL	40±14	40±15	40±14	40±14	39±13	<.001
LDL	85±35	93±37	108±44	85±36	79±30	<.001
Cholesterol	156±46	165±49	183±55	155±47	148±41	<.001
Triglycerides	127 [90,187]	122 [85,182]	146 [103,223]	126 [89,185]	129 [91,187]	<.001
Number of prescribed medications						
At time of transition to dialysis	8[5,14]	6[3,11]	6[3,11]	8[5,12]	11[7,17]	<.001
6 months After transition to dialysis	7[4,10]	5[2,8]	7[5,11]	4[2,8]	8[5,12]	<.001
Initial Dialysis Modality (%)						0.001
Hemodialysis	90	90	89	89	89	
Peritoneal Dialysis	6	6	7	5	7	
Other/Unknown	4	4	4	6	4	
Initial Access type (%)						<.001
AV Fistula/ AV Graft	26	23	21	22	30	
CVC	66	69	70	72	62	
Other/Unknown	8	8	9	7	9	
AKI diagnosis in year prior to transition (%)	25	26	28	31	23	<.001
Any VA or CMS physician nephrology visits in the year prior to	72	68	67	71	75	<0.001

© 2018 Streja E et al. *JAMA Network Open.*

transition (%)						
# VA or CMS physician nephrology visits in the year prior to transition	4 [0,8]	3 [0,7]	3[0,7]	3 [0, 8]	4 [1,8]	<0.001
Any VA nephrology visits in the year prior to transition (%)	39	34	31	34	45	<0.001
# VA nephrology visits in the year prior to transition	0[0,3]	0 [0,2]	0 [0,1]	0 [0,2]	0 [0,4]	<0.001
Any CMS physician nephrology visits in the year prior to transition (%)	40	40	43	45	39	<0.001
# CMS physician nephrology visits in the year prior to transition	0[0,4]	0 [0, 4]	0 [0,4]	0 [0,5]	0 [0,4]	<0.001

Abbreviations: AV; arteriovenous, CVC; central venous catheter, eGFR; estimated glomerular filtration rate, ESRD; end-stage renal disease, CCI; Deyo Charlson Comorbidity Index, ISHD; Ischemic Heart Disease; MI; myocardial infarction, CHF; congestive heart failure, PVD; peripheral vascular disease, COPD; chronic obstructive pulmonary disease, VA; Veteran Administration, CMS; Centers for Medicare and Medicaid Services.

Data presented as proportion, mean ± standard deviation or median [interquartile range] where appropriate, and compared between groups using ANOVA, Kruskal-Wallis or chi-square tests, as appropriate.

© 2018 Streja E et al. *JAMA Network Open.*

eTable 5. Event Rate and Hazard Ratios for the Association of Statin Pre and Post Transition Groups (ref: None Before or After ESRD) With A) All-Cause Mortality and B) Cardiovascular Mortality Over 12-Months of Follow-up

A)

**All-Cause Mortality**						
Group	Events	Crude Rate per 100 person-years [95%CI]	Unadjusted		Adjusted	
			HR[95%CI]	p-value	HR[95%CI]	p-value
**12-month**						
Total (n=25,424)	4910 (19%)	23.4[22.7,24.0]				
None Before or After ESRD (n=9,691)	1909 (20%)	24.0[23.0,25.1]	Ref		Ref	
None Before ESRD but Statins After ESRD (n=1,435)	261 (18%)	21.0[18.4,23.5]	0.87[0.77,0.99]	0.04	0.83[0.73,0.94]	0.004
Statins Before ESRD but None After (n=2,369)	558 (24%)	30.3[27.8,32.8]	1.26[1.15,1.39]	<.001	1.05[0.95,1.15]	0.32
Statins Before and After ESRD (n=11,936)	2182 (18%)	21.9[20.9,22.8]	0.91[0.86,0.97]	0.003	0.76[0.71,0.81]	<.001

B)

**Cardiovascular Mortality**						
Group	Events	Crude Rate per 100 person-years [95%CI]	Unadjusted		Adjusted	
			HR[95%CI]	p-value	HR[95%CI]	p-value
**12-month**						
Total (n=25,424)	1634 (6%)	8.0[7.6,8.4]				
None Before or After ESRD (n=9,691)	600 (6%)	7.8[7.1,8.4]	Ref		Ref	
None Before ESRD but Statins After ESRD (n=1,435)	76 (5%)	6.2[4.8,7.6]	0.80[0.63,1.02]	0.07	0.71[0.56,0.91]	0.01
Statins Before ESRD but None After (n=2,369)	174 (7%)	9.8[8.3,11.2]	1.26[1.07,1.49]	0.01	0.99[0.84,1.18]	0.95
Statins Before and After ESRD (n=11,936)	784 (7%)	8.1[7.5,8.6]	1.04[0.93,1.16]	0.48	0.81[0.72,0.90]	<.001

Adjusted covariates: age, gender, race, and ethnicity as well as the following comorbidities: CCI, diabetes, ASCVD (defined as presence of MI, PVD, or ISHD), atrial fibrillation, CHF and cerebrovascular disease.

© 2018 Streja E et al. *JAMA Network Open.*

eTable 6. Sensitivity Analysis Showing Associations of Statin Continuation vs. Discontinuation With All-Cause Mortality Risk After Additional Adjustment for Other Factors

Model	N	p-value	HR (95%CI)
Adjusted	14,298	<0.001	0.72 (0.66, 0.79)
Adjusted +any nephrology visits in year prior to dialysis (yes/no)	14,298	<0.001	0.73 (0.67, 0.80)
Adjusted +any CMS nephrology visit in year prior to dialysis (yes/no)	14,298	<0.001	0.73 (0.66, 0.80)
Adjusted +any VA nephrology visit in year prior to dialysis (yes/no)	14,298	<0.001	0.72 (0.66, 0.79)
Adjusted + # nephrology visits	14,298	<0.001	0.73 (0.66, 0.80)
Adjusted + # VA nephrology visits	14,298	<0.001	0.74 (0.67, 0.81)
Adjusted + # CMS nephrology visits	14,298	<0.001	0.72 (0.66, 0.79)
Adjusted + access type	14,298	<0.001	0.74 (0.68, 0.82)
Adjusted +AKI prior to transition	14,298	<0.001	0.72 (0.66, 0.79)
Adjusted + #medications at baseline	14,298	<0.001	0.71 (0.65, 0.78)
Adjusted + #medications at baseline and 6 months post-ESRD	14,298	<0.001	0.70 (0.64, 0.77)
Adjusted + Allopurinol Discontinuation	14,298	<0.001	0.73 (0.67, 0.80)
Adjusted + Beta Blocker Discontinuation	14,298	<0.001	0.73 (0.66, 0.80)
Adjusted + Multivitamin Discontinuation	14,298	<0.001	0.72 (0.66, 0.80)
Adjusted + RAAS Inhibitor Discontinuation	14,298	<0.001	0.73 (0.67, 0.80)
Adjusted + # medication groups discontinued post-transition	14,298	<0.001	0.73 (0.66, 0.80)
Adjusted + Smoking Status*	12,698	<0.001	0.73 (0.66, 0.80)
Adjusted + estimated glomerular filtration rate at transition*	13,936	<0.001	0.72 (0.65, 0.79)
Adjusted + lipids (cholesterol, HDL, LDL, triglycerides)*	9,322	<0.001	0.70 (0.62, 0.80)

Adjusted covariates: age, gender, race, and ethnicity as well as the following comorbidities: CCI, diabetes, ASCVD (defined as presence of MI, PVD, or ISHD), atrial fibrillation, CHF and cerebrovascular disease.

**Limited to patients who went to the VA for outpatient visits and had data on covariate of interest in the year prior to transition*

© 2018 Streja E et al. *JAMA Network Open.*

eTable 7. Sensitivity Analysis Showing Associations of Statin Continuation vs. Discontinuation With All-Cause Mortality Risk After Propensity Score Matching, Adjustment, and Stratification

		**All-Cause Mortality**				**Cardiovascular Mortality**			
		Unadjusted		Adjusted		Unadjusted		Adjusted	
	N	HR[95%CI]	p-value	HR[95%CI]	p-value	HR[95%CI]	p-value	HR[95%CI]	p-value
Matching	4724	0.68[0.60,0.77]	<.0001	0.66[0.58,0.75]	<.001	0.80[0.64,0.99]	0.04	0.77[0.62,0.96]	0.02
Adjustment *	14298	0.73[0.66,0.80]	<.0001	0.72[0.66,0.79]	<.001	0.82[0.70,0.97]	0.02	0.81[0.69,0.96]	0.01
Tertile 1	4714	0.76[0.65,0.88]	<.001	0.77[0.66,0.90]	<.001	0.81[0.62,1.07]	0.13	0.81[0.61,1.06]	0.13
Tertile 2	4870	0.68[0.58,0.80]	<.0001	0.67[0.57,0.79]	<.001	0.80[0.61,1.05]	0.10	0.77[0.58,1.01]	0.06
Tertile 3	4714	0.73[0.61,0.87]	<.001	0.72[0.61,0.86]	<.001	0.87[0.64,1.19]	0.39	0.86[0.63,1.18]	0.35

Adjusted covariates: age, gender, race, and ethnicity as well as the following comorbidities: CCI, diabetes, ASCVD (defined as presence of MI, PVD, or ISHD), atrial fibrillation, CHF and cerebrovascular disease.

© 2018 Streja E et al. *JAMA Network Open.*

eTable 8. Medication Groups Ever Prescribed in the Year Pre and Post Transition to Dialysis in Statin Continuation and Statin Discontinuation Patients

	One year Pre-ESRD transition			One year Post-ESRD transition		
	Statin Discontinued	Statin Continued	Std Diff	Statin Discontinued	Statin Continued	Std Diff
**Medication ever use N(%)**	2362	11936		2362	11936	
**Allopurinol**	499 (21)	2938 (25)	0.08	462 (20)	2943 (25)	0.12
**Aspirin**	553 (23)	3664 (31)	0.16	477 (20)	3817 (32)	0.27
**Beta blocker**	1815 (77)	9571 (80)	0.08	1762 (75)	9875 (83)	0.20
**Multivitamin (*primarily VA data)**	279 (12)	1960 (16)	0.13	570 (24)	4013 (34)	0.21
**Anticoagulants**	366 (16)	2652 (22)	0.17	302 (13)	2667 (22)	0.25
**Coumadin**	271 (11)	1397 (12)	0.01	291 (12)	1804 (15)	0.08
**Thrombolytics**	12 (1)	71 (1)	0.01	29 (1)	230 (2)	0.06
**Antiplatelet**	426 (18)	2375 (20)	0.05	429 (18)	2932 (25)	0.16
**Erythropoietin Stimulating Agents**	406 (17)	2986 (25)	0.19	339 (14)	2651 (22)	0.20
**Opioids**	839 (36)	4900 (41)	0.11	915 (39)	5562 (47)	0.16
**Non-opioid analgesics**	330 (14)	1987 (17)	0.07	310 (13)	2176 (18)	0.14
**Antidepressants**	639 (27)	3546 (30)	0.06	707 (30)	4032 (34)	0.08
**Digitalis**	122 (5)	500 (4)	-0.05	123 (5)	574 (5)	-0.02
**Alpha blocker**	878 (37)	4903 (41)	0.08	802 (34)	4855 (41)	0.14
**Calcium channel blocker**	1611 (68)	8869 (74)	0.14	1497 (63)	8534 (72)	0.17
**Antianginals**	623 (26)	3683 (31)	0.10	584 (25)	3862 (32)	0.17
**Antiarrhythmics**	106 (4)	547 (5)	0.00	165 (7)	897 (8)	0.02
**Vasodilator Antihypertensive**	694 (29)	4085 (34)	0.10	640 (27)	4075 (34)	0.15
**Thiazides**	461 (20)	2631 (22)	0.06	287 (12)	1744 (15)	0.07
**Loop diuretics**	1613 (68)	9131 (77)	0.18	1388 (59)	8555 (72)	0.27
**K sparing diuretics**	178 (8)	800 (7)	-0.03	109 (5)	531 (4)	-0.01
**RAAs inhibitors**	1329 (56)	6934 (58)	0.04	1112 (47)	6606 (55)	0.17
**Bicarbonate**	299 (13)	1859 (16)	0.08	237 (10)	1640 (14)	0.11
**Insulin**	1019 (43)	5722 (48)	0.10	989 (42)	5913 (50)	0.15
**Oral Hypoglycemic Medications**	697 (30)	3316 (28)	-0.04	549 (23)	2822 (24)	0.01
**Thyroid supplements**	346 (15)	1833 (15)	0.02	363 (15)	2004 (17)	0.04
**Iron**	490 (21)	3603 (30)	0.22	469 (20)	3636 (30)	0.25
**Calcium**	189 (8)	1301 (11)	0.10	233 (10)	1601 (13)	0.11
**Citrate**	60 (3)	474 (4)	0.08	35 (1)	313 (3)	0.08
**25 Vitamin D**	306 (13)	2027 (17)	0.11	306 (13)	2102 (18)	0.13
**Active Vitamin D**	696 (29)	4520 (38)	0.18	598 (25)	4192 (35)	0.21
**Sevelamer**	239 (10)	1656 (14)	0.12	622 (26)	3896 (33)	0.14
**Lanthanum**	29 (1)	266 (2)	0.08	94 (4)	691 (6)	0.08
**Calcium Acetate**	351 (15)	2535 (21)	0.17	604 (26)	4533 (38)	0.27
**Centrally Acting Alpha Agonists**	500 (21)	2605 (22)	0.02	451 (19)	2489 (21)	0.04

© 2018 Streja E et al. *JAMA Network Open.*

eTable 9. Discontinuation of Other Medications

	Statin Discontinued	Statin Continued
	2362	11936
**Medication**	**N/total and (%) Continued Medication**	**N/total and (%) Continued Medication**
Allopurinol	182/217 (84)	1744/1838 (95)
Beta Blockers	777/867 (90)	6374/6528 (98)
RAAS Inhibitors	336/513 (66)	2850/3493 (82)
Median (IQR): Total Medication Groups Discontinued *from Table S7	1 (1, 2)	1 (0,1)

© 2018 Streja E et al. *JAMA Network Open.*

eTable 10. Studies Investigating Statin Use in Dialysis Patients

	**4D**[8]	**AURORA**[7]	**ESRD Subgroup from SHARP**[2]
Number of patient	1,255	2,776	3,023
Study design	RCT	RCT	RCT
Statin initiation	De novo	De novo	De novo
Average follow-up, years	4	3.9	4.9
Duration on dialysis	8.6 months	3.5 years	Not reported
Primary outcome definition and results	Composite death from CV causes, nonfatal MI and stroke<br><br>RR 0.92 (95%CI 0.77-1.10)	Composite death from CV causes, non-fatal MI and stroke<br><br>HR 0.96 (95%CI 0.84-1.11)	Nonfatal MI or coronary death, non-hemorrhagic stroke, or arterial revascularization procedure<br>HR 0.90 (95%CI 0.75-1.08)
All-cause mortality	RR 0.93 (95% CI 0.79-1.08)	HR 0.96 (95% CI 0.86-1.07)	Not reported for ESRD but the whole study<br>HR 1.02 (95% CI 0.94-1.11)
CV mortality	RR 0.81 (95% CI 0.64-1.03)	HR 1.00 (95% CI 0.85-1.16)	Not reported for ESRD patients separately but the whole study<br>HR 0.93 (95% CI 0.80-1.07)

Abbreviations: 4D, the Deutsche Diabetes Dialyse Studie; AURORA, A study to evaluate the Use of Rosuvastatin in subjects On Regular hemodialysis: an Assessment of survival and cardiovascular events; SHARP, Study of Heart And Renal Protection; RCT, randomized controlled trial; CKD, chronic kidney disease; ESRD, end-stage renal disease; CV, cardiovascular; MI, myocardial infarction; RR, relative risk; HR, hazard ratio; CI, confidence interval

© 2018 Streja E et al. *JAMA Network Open.*

eTable 11. Risk Ratios for Statin Continuation, Hazard Ratios for All-Cause Mortality, and Estimate E-Values for Covariates Included in Fully Adjusted Model

	Risk Ratio of Statin Continuation					Adjusted Hazard Ratio with All-Cause Mortality				
Variable	p-value	Risk Ratio	95% Risk Ratio Confidence Limits		inverse RR if <1	p-value	Hazard Ratio	95% Hazard Ratio Confidence Limits		inverse HR if <1
Statin Continuation	n/a	n/a	n/a	n/a	n/a	<.0001	0.72	0.66	0.79	
Age (per 1 year)	0.4	1.00	1.00	1.00	1.00	<.0001	1.02	1.02	1.03	1.02
Gender (Female vs. Male)	0.4	1.02	0.97	1.06	1.02	0.7	0.96	0.76	1.20	1.04
African American Race (reference=White)	0.004	1.03	1.01	1.05	1.03	<.0001	0.72	0.64	0.80	1.39
Other Race (reference=White)	0.4	0.99	0.96	1.02	1.01	0.7	0.97	0.80	1.17	1.04
Hispanic Ethnicity (reference= non-Hispanic)	0.3	1.02	0.99	1.05	1.02	0.08	0.86	0.72	1.02	1.17
Charlson Comorbidity Index	<.0001	0.99	0.99	0.99	1.01	<.0001	1.09	1.07	1.11	1.09
Diabetes	0.008	0.97	0.96	0.99	1.03	0.10	0.92	0.84	1.02	1.08
ASCVD	0.02	0.98	0.96	1.00	1.02	<.0001	1.26	1.13	1.42	1.13
Atrial Fibrillation	0.6	1.01	0.98	1.03	1.01	0.0002	1.20	1.09	1.32	1.09
Congestive Heart Failure	0.4	0.99	0.97	1.01	1.01	<.0001	1.29	1.18	1.42	1.18
Cerebrovascular Disease	0.003	0.97	0.96	0.99	1.03	0.5	1.03	0.94	1.12	1.03

© 2018 Streja E et al. *JAMA Network Open.*

ASCVD= Atherosclerotic Cardiovascular Disease and includes myocardial infarction, peripheral vascular disease, and ischemic heart disease

© 2018 Streja E et al. *JAMA Network Open.*
